# Modeling relationships between rhythmic processes and neuronal spike timing

**DOI:** 10.1152/jn.00423.2021

**Published:** 2022-07-20

**Authors:** Pamela D. Rivière, Gabriel Schamberg, Todd P. Coleman, Lara M. Rangel

**Affiliations:** ^1^Department of Cognitive Science, University of California, La Jolla, California; ^2^Massachusetts Institute of Technology, Picower Institute for Learning and Memory, Cambridge, Massachusetts; ^3^Department of Bioengineering, University of California, La Jolla, California

**Keywords:** hippocampus, interneurons, rhythms, spike-phase relationships, statistical modeling

## Abstract

Neurons are embedded in complex networks, where they participate in repetitive, coordinated interactions with other neurons. Neuronal spike timing is thus predictably constrained by a range of ionic currents that shape activity at both short (milliseconds) and longer (tens to hundreds of milliseconds) timescales, but we lack analytical tools to rigorously identify these relationships. Here, we innovate a modeling approach to test the relationship between oscillations in the local field potential (LFP) and neuronal spike timing. We use kernel density estimation to relate single neuron spike timing and the phase of LFP rhythms (in simulated and hippocampal CA1 neuronal spike trains). We then combine phase and short (3 ms) spike history information within a logistic regression framework (“phaseSH models”), and show that models that leverage refractory constraints and oscillatory phase information can effectively test whether—and the degree to which—rhythmic currents (as measured from the LFP) reliably explain variance in neuronal spike trains. This approach allows researchers to systematically test the relationship between oscillatory activity and neuronal spiking dynamics as they unfold over time and as they shift to adapt to distinct behavioral conditions.

**NEW & NOTEWORTHY** Statistical models that incorporate neural spiking history and relationships to the phase of ongoing oscillations in the local field potential robustly capture and predict neuronal engagement in rhythmic processes. These models constitute a powerful tool to systematically test explicit hypotheses regarding the specific rhythmic currents that constrain neural spiking activity over time and during different behaviors.

## Graphical abstract

**Figure:**
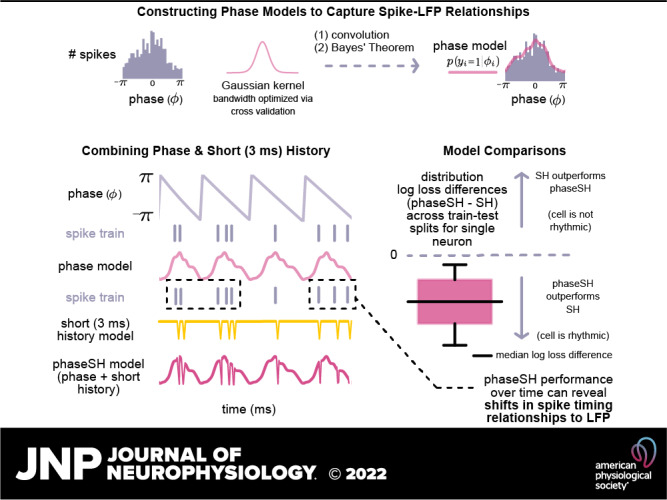

## INTRODUCTION

The neural code crucially depends on spike timing. In the brain, variations in spiking patterns over time convey information about the external world (1), instigate (and register) changes within the body (2), and engage distinct behavioral states (2–4). A major aim in neuroscience is the quantification of temporal regularity in these spike trains and the identification of its underlying generators. Establishing relationships between temporal patterns of spiking activity and their neural drivers is challenging, however, and requires methods that can *1*) describe temporal fluctuations and *2*) evaluate possible neural mechanisms responsible for these fluctuations. Individual neurons in the central nervous system are embedded in complex networks, where they receive inputs from a variety of both distally located and neighboring neurons. Individual neurons additionally generate spontaneous intrinsic ionic currents that interact with these input sources. Currently, few quantitative methods are designed to systematically assess the contribution of specific rhythmic currents in shaping single-neuron spike timing. Here, we provide a powerful statistical modeling approach that allows us to weigh the relative contributions of refractory and rhythmic current influences on neuronal spike timing, predict moment-to-moment variations, quantify the uncertainty of our predictions, and assess the goodness-of-fit of our models.

Neuronal activity obeys constraints at multiple timescales. Action potentials (spikes) are large-amplitude, short-lived (<1 ms) neuronal membrane voltage deflections. These are followed by transient periods of hyperpolarization, during which neurons are less likely or unable to emit further spikes (5–7). At longer timescales, regularly occurring ionic currents create phasic temporal windows during which an individual neuron is more or less primed to produce spikes (8, 9). These currents can arise spontaneously in the absence of synaptic inputs (10, 11), or they may result from repetitive, coordinated network interactions. Notably, these longer-timescale ebbs and flows in subthreshold membrane potentials, whether intrinsically or synaptically generated, superimpose extracellularly to produce neural oscillations in the local field potential (LFP) (12, 13). Oscillations in the LFP thus reflect a mixture of intrinsic and synaptic currents shaping single-neuron activity, but the degree to which these combined currents influence spike timing remains uncertain.

In the hippocampus, a brain region extensively studied in the context of learning and memory, pyramidal cell and inhibitory interneuron spiking is often rhythmically organized in the theta (4–12 Hz) and low gamma (35–55 Hz) frequency ranges (14–17), and evidence suggests that interneurons powerfully contribute to periodicity in the hippocampal LFP (18, 19). Rhythmic temporal structure in interneuron spike trains arises from both neuronal resonance [i.e., intrinsic cellular properties that give rise to preferential responses at specific frequency ranges and spontaneous membrane oscillations, (20, 21)] as well as interactions with a range of intra-hippocampal (22, 23), cortical (22–25), and subcortical (26–28) inputs. Thus, hippocampal interneuron spiking is temporally constrained at both short timescales (i.e., milliseconds) due to refractoriness, and at longer theta and gamma rhythmic timescales (i.e., tens to hundreds of milliseconds) due to a combination of intrinsic and synaptic current fluctuations. Whereas the influence of the afterhyperpolarization on neuronal spiking is well known, and the temporal structure of this influence has been characterized via statistical models (29, 30), we currently lack the tools to evaluate the effect that longer-duration rhythmic constraints exert on spike timing. Moreover, there are few existing methods for assessing short- and long timescale oscillatory influences together to provide a holistic view of the temporal constraints that can shape neuronal spiking. Here, we deploy a series of statistical models that leverage spike history information as well as LFP rhythms to model the rhythmic temporal structure of both simulated and hippocampal inhibitory interneuron spike trains.

Our approach is grounded in the use of generalized linear models (GLMs) and maximum likelihood estimation (31), which have been used extensively to account for the absolute and relative refractory periods following a spike occurrence (29, 30, 32, 33). Typically, these models use logistic regression to predict the probability of spiking from the preceding spike history. Following previous work (29, 30, 32, 33), we constructed a short history model (“SH model”), which predicted upcoming spiking according to spike history in the preceding 3 ms. Beyond capturing the absolute afterhyperpolarization constraint, we also modeled the relationship between spiking and a longer-duration history (250 ms, “LH model”) to detect patterns in spike emissions that cycled along hundreds of milliseconds at most. This LH model allowed us to flexibly capture structure in spike trains at exquisitely fine temporal resolution without prior assumptions about specific factors that contributed to the structure (e.g., refractoriness, spike-rate adaptation, rhythmicity at various frequency ranges). In this way, LH models behaved much like sliding-window autocorrelations or spectral analysis of spike trains (34), but applied in a predictive (rather than exclusively descriptive) setting. Despite their descriptive and predictive abilities, LH models do not by themselves disambiguate the various possible neural mechanisms underlying temporal structure in spike trains.

To explicitly test hypotheses regarding the presence of rhythmicity in spike trains, we developed a model that could represent rhythmic currents as measured from the LFP, and also quantify their relationship to neuronal spike trains. In the work that follows, we present strategies for *1*) representing spiking relationships to the phase of a particular LFP rhythm using kernel density estimation (KDE) and *2*) combining LFP phase information and neuronal refractory constraints on spiking. First, we used KDE to directly model the likelihood of LFP phases given observed spiking, and subsequently derived the posterior probability of spiking over time given a phase time series. Since phase models could not capture short timescale refractory constraints on spiking, and SH models were insensitive to long timescale structure, we innovatively combined phase and short history information in a logistic regression framework (“phaseSH models”). These comprehensive models were able to effectively capture and specify short (refractory) and long (rhythmic) timescale influences in simulated spike trains and hippocampal CA1 neurons, which addressed the major limitations of each model independently. Moreover, since our models specifically assess the probability of spiking relative to phase, they provide information about the reliability of these relationships over time that cannot be obtained from traditional measures of spike-phase relationships based on circular averages (e.g., the Rayleigh statistic). We additionally compared phaseSH model performance against that of phaseLH models (GLMs containing phase and long history as predictors), to test whether including long history provided additional information to better predict held-out spike trains. In our data set, most spike trains were better predicted by the phaseSH models, suggesting that long history did not add information above and beyond phaseSH models in these cases. The model comparisons described in this work provide a new paradigm for rigorously assessing the contribution of rhythmic processes to single-neuron spiking dynamics.

## MATERIALS AND METHODS

### Experimental Design: Behavioral Paradigm

All procedures involving animals were carried out according to guidelines set forth by the National Institute of Health, with approval from the Institutional Animal Care and Use Committee (IACUC) in Boston University (Approval Number: 13-057). Experimental procedures have been previously described at length in the study by Rangel et al. (35) and will be briefly reviewed here.

We aimed to elicit multiple stereotyped and behaviorally controlled instances of “associative memory processing” during a window of time in which rats had to integrate information about odor cues and the context in which they were presented to produce an appropriate, rewarded response.

#### Odor sampling epoch.

The data analyzed in this study were recorded from the CA1 subregion of the hippocampus in rats performing a context-guided associative memory task (35, 36). Rats learned that odors in a pair were differentially rewarded as a function of the context in which they appeared. Contexts were differentiated according to their spatial position in the room as well as the texture and coloring of material wrapped around the context’s surface. During a given trial, rats were allowed access to one context at a time, and odor delivery within an odor port (start time of each trial) was triggered 250 ms after the rat poked his nose into that odor port. Odor reward contingencies remained constant throughout the experiment, and odor delivery locations were pseudorandomized across odor ports within a context. Two pairs of odors were used for each session, with the first block (48 consecutive trials) featuring *odors A* and *B*, and the second block consisting of *odors C* and *D*, for a total of 96 trials per session. Interneuron data recorded from each block of odors was analyzed separately, given the potential differences in spiking temporal structure driven by distinct contexts. Pyramidal cell data were analyzed separately for each odor-position combination since these neurons have previously been shown to spike selectively for specific positions and odor-position combinations in this task (35).

#### Correct trials.

To receive a water reward, rats maintained a nose poke in the correct odor port (the one containing the rewarded odor within that context) for 1,250 ms following the odor onset, after which a water droplet was delivered to a well directly below the odor port. If the rats initially poked their nose into the incorrect port, they had up to 1,250 ms following odor onset to remove their snout, after which they were able to poke into the correct odor port and maintain the nose poke. For our phase modeling efforts, all intervals were restricted to the 1,250 ms intervals following odor onset in which rats maintained their nose in the odor port containing the correct odor before receiving a reward. We refer to this interval as the “odor sampling epoch.” The first time point assessed by our models begins at the time of odor presentation, with the preceding 250 ms serving solely as spike history for the first 250 time samples of the odor-sampling interval.

#### Incorrect trials.

If the rat failed to end the nose poke 1,250 ms following odor onset in the incorrect odor port, a buzzer would sound and no reward would be delivered. Recordings began only after rats had successfully achieved a 75% performance criterion. Incorrect trials are consequently infrequent, and we excluded them from this study.

#### Approach epoch.

We also separately fit models to data drawn from a behaviorally distinct epoch: the running approach to the odor port. To create these approach epochs, we selected the 1,500 ms interval with the highest median velocity preceding a correct odor sampling trial. As we did for the odor sampling epoch, phase models are fit to data from a continuous 1,250-ms period, with the preceding 250 ms serving as spike history for the first 250 time samples of the approach intervals.

### Data Acquisition

We carried out high-density extracellular tetrode recordings from the CA1 subregion of the hippocampus and extracted single-unit and local field potential (LFP) data. The Omniplex Neural Data Acquisition system (Plexon) amplified (4,000–8,000 times) and digitized (40 kHz) signals.

#### Single units.

Putative interneurons were isolated according to waveform features: mean firing rate (≥5 Hz), mean width at half-maximum of waveform (<150 μs), and mean temporal offset from peak to trough (<350 μs) (18, 37).

#### Local field potentials.

LFPs were bandpass-filtered in the theta (4–12 Hz) and low gamma (35–55 Hz) ranges using a third-order Butterworth filter. We then obtained instantaneous phase estimates by computing the arctangent of the filtered signal’s complex Hilbert transform. Frequency range selection was based on the observable frequency bands in average spectrograms during the odor sampling epoch (35).

### Data Preparation

In the present study, we examined interneuron spiking and CA1 local field potentials (LFPs) for single correct trials, each consisting of 1,500 1-ms time samples (250 ms before odor stimulus delivery, 1,250 ms after odor stimulus delivery). Time samples occurring within trials are consecutively sampled (sampling frequency = 1,000 Hz), while time samples between trials are separated by longer (and variable) stretches of time. Each interneuron spike train was analyzed with respect to the simultaneously recorded CA1 LFP, acquired from the same tetrode that collected single-unit spikes. To test the generalizability of trained models, we partitioned trial labels into train (50%) and test sets (50%). We generated a total of 20 train-test splits for each neuronal spike train. Consecutive time samples within a trial retained their temporal structure.

#### History variables.

**Figure 1.**
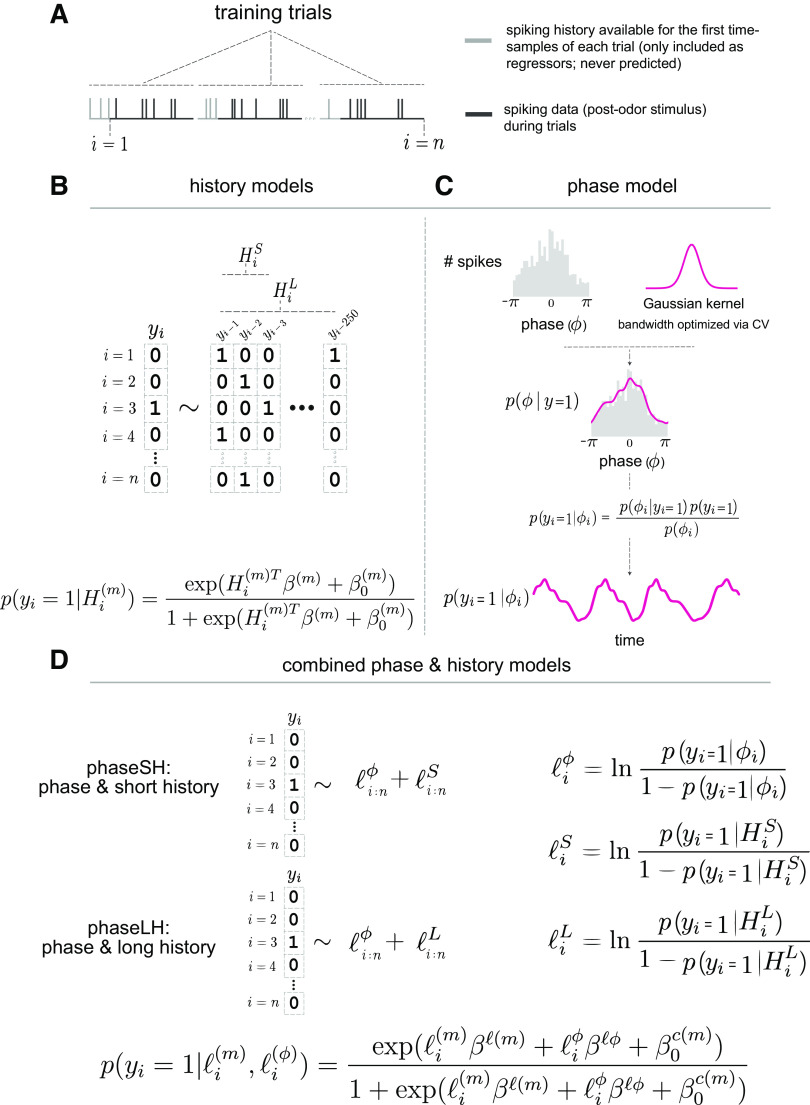
Data structure and model specification. *A*: data are from a previously published study, in which rats successfully associated odors with a rewarded context. All analyzed intervals correspond to 1,250 ms epochs (spikes in dark gray) with a preceding 250 ms serving solely as history regressors for the first time samples of the behavioral epochs (spikes in light gray). *B*: long (LH) and short history (SH) models, generated via logistic regression. The target consists of a neuron spike (or the lack thereof), *y_i_*, for each time sample *i*. Predictors are either the 3 ms (short history, $H_{i}^{S}$) or the 250 ms (long history, $H_{i}^{L}$) preceding the current time sample. LH model coefficient estimates are uniquely subject to L2 regularization. *C*: phase model, generated via kernel density estimation (KDE). We perform cross validation to optimize the bandwidth of a Gaussian kernel. The resulting estimator represents the probability of a phase given that a spike has occurred, and this estimator can then be used to predict the probability of spiking over time, as a function of a phase time series. *D*: models combining a phase and history predictor. Each of these predictors is represented as the log-odds of the phase-based probabilities of spiking, $l^{\phi }$, and the log odds of history-based probabilities of spiking, $l^{\left(m\right)}$, respectively.

During the odor sampling epoch, the odor that the rat used to make a decision (to stay or move to the next odor port) was made available 250 ms after the nose poke. In the first 250 ms of the trial, the rat’s behavior is the same (stationary) as its behavior for the rest of a correct trial, the only difference being the availability of the odor. Given this, we use the first 250 ms of the trial as the history for the first time samples post-odor release. The 250 ms at the very beginning of each trial were only included in the models as spiking history regressors; they never served as target variables (Fig. 1*A*). Fitting history models to data from the approach epochs proceeded identically.

### Data Simulations

We produced synthetic data to simulate four distinct “types” of spike trains, each with unique ground-truth dependencies that governed spike probability. These simulated spike trains exhibited: *1*) no temporal modulation/“atemporal” with no refractory period and no relationship with respect to a simulated phase time series oscillating at 8 Hz, *2*) short timescale temporal modulation/“refractory non-rhythmic” with a refractory period (lasting 3 ms postspike) and no relationship with respect to a simulated phase time series oscillating at 8 Hz, *3*) long timescale modulation/“non-refractory rhythmic” with no refractory period but a relationship with respect to a simulated phase time series oscillating at 8 Hz, and 4) both short and long timescale modulation/“refractory rhythmic” with a refractory period (lasting 3 ms postspike) and a relationship with respect to a simulated phase time series oscillating at 8 Hz.

We simulated 48 time series, each spanning 1,500 ms (identical in length and approximate number to trials in our behavioral task, where the first 250 ms of each trial serve exclusively as history predictors for the early time samples of each odor sampling interval, Fig. 1*A*). For each time series, we created a time-matched vector of probability densities indicating which phases were most likely to coincide with spikes. For simulated spike trains that had no phase relationship, each cycle of the probability vector consisted of a flat, uniform distribution whose value corresponded to the average probability of spiking. For simulated spike trains governed by a phase relationship, each cycle of the probability vector consisted of a von Mises distribution peaking at 0 radians, with an inverse variance of 2 (equivalent to a Gaussian kernel bandwidth of 0.5). Phase cycles were each 125 ms long, simulating an 8 Hz oscillation. This probability vector thus represented the probability of spiking given the phase of an 8 Hz oscillation. To equip simulated spike trains with a refractory period, we forced the model probabilities generated earlier to adjust to 0.00001 (essentially zero) in the three time samples immediately following a sampled spike.

To sample spiking events from these ground truth probabilities, we used a single-trial binomial distribution (software: Python Numpy, random.binom), which produced a spike (1) or not a spike (0) according to the probability value at that particular time sample. Once the simulated data had been generated, each simulated spike train underwent history and phase model-fitting as described in sections *Constructing History Models*, *Learning Phase Representations*, and *Models Combining Phase and History Information*. To test the degree of divergence between model-based probabilities and ground truth probabilities, we calculated the Kullback–Leibler (KL) divergence,

(*1*) $$D(p_{i}||q_{i})=\;p_{i}\;\log_{2}\frac{p_{i}}{q_{i}}+(1-\;p_{i})\;{\log\;}_{2}\frac{\left(1-\;p_{i}\right)}{\left(1-\;q_{i}\right)}$$

where $p_{i}$ and $q_{i}$ are the ground truth and modeled probability of spiking at time sample *i*, respectively. This value can be interpreted as the cost, in bits, incurred when modeling the distribution of spikes using the model-based probabilities instead of the ground truth.

We computed the KL divergence at each time sample to compare the ground truth to a given model’s predictions. We then computed the average of all the KL divergences across time, to provide an overall sense for the degree of misalignment between the ground truth and model-based predictions throughout the entirety of the time series. A smaller average KL divergence indicates increased alignment between the ground truth and the model.

### Constructing History Models

We constructed models that used the history of spiking over long or short timescales. For short history models (“SH models”), we reasoned that using 3 ms of history would capture both the absolute and initial segments of the relative refractory periods that neurons undergo immediately after emitting an action potential. In contrast, the long history model (“LH model”) can reflect various temporal constraints on spiking over a more extended past. Consequently, for neurons in the hippocampus, the history should also encode the periodic structure of spiking due to oscillatory current modulation, if it exists. To test whether long history could capture the periodic distribution of spikes over time, we set the long history interval to begin 250 ms before current time sample *i*, equivalent to a full period of the lowest frequency (4 Hz) within the theta 4–12 Hz frequency range, and additionally able to capture any rhythmic current influences at faster frequencies.

To generate probabilities of spiking given the long and short histories separately, we fit logistic regression models, according to the expression below,

(*2*) $$p\left(y_{i}=1\text{|}H_{i}^{\left(m\right)}\right)=\;\frac{\exp\;(H_{i}^{\left(m\right)T}\beta^{\left(m\right)}+{\text{ β}}_{0}^{\left(m\right)})}{1+\;\exp\;(H_{i}^{\left(m\right)T}\beta^{\left(m\right)}+{\text{ β}}_{0}^{\left(m\right)})},$$

where $y_{i}$ corresponds to spiking at time sample *i* and $H_{i}^{\left(m\right)}$ is a vector that represents history, long (with dimensions: 1 × 250) or short (with dimensions: 1 × 3), for that time sample. In models that commit to including either long history or short history parameters, *m* is replaced by *L* for long history, or *S* for short history (Fig. 1*A*). For ease of notation, we define the vectors ${\tilde{\bar{H}}}_{i}^{\left(m\right)}={\left[1\;H_{i}^{\left(m\right)}\right]}^{T}$ and ${\tilde{\bar{\beta }}}^{\left(m\right)}={\left[{\text{β}}_{0}^{\left(m\right)}\;\beta^{\left(m\right)}\right]}^{T}$, such that ${\tilde{\bar{H}}}_{i}^{\left(m\right)T}{\tilde{\bar{\beta }}}^{\left(m\right)}=H_{i}^{\left(m\right)T}\beta^{\left(m\right)}+\;\beta_{0}^{\left(m\right)}$. To estimate the coefficient vector ${\tilde{\bar{\beta }}}^{\left(m\right)}$, we find the vector that minimizes the normalized negative log likelihood, NNLL, given by

(*3*) $$\text{ NNLL}({\tilde{\bar{\beta }}}^{\left(m\right)})=\frac{1}{n}\sum_{i=1}^{n}-y_{i}\left({\tilde{\bar{H}}}_{i}^{\left(m\right)T}{\tilde{\bar{\beta }}}^{\left(m\right)}\right)+\log\;(1+e^{{\tilde{\bar{H}}}_{i}^{\left(m\right)T}{\tilde{\bar{\beta }}}^{\left(m\right)}}).$$

For short history models, we fit a coefficient vector ${\tilde{\bar{\beta }}}^{\left(S\right)}$ containing four parameters (${\tilde{\bar{\beta }}}^{\left(S\right)}=\;\beta_{0}^{\left(S\right)},\beta_{1}^{\left(S\right)},\;\beta_{2}^{\left(S\right)},\beta_{3}^{\left(S\right)}$), with one parameter for the baseline spike rate and one for each millisecond of short history. In contrast, long history models require fitting a coefficient vector ${\tilde{\bar{\beta }}}^{\left(L\right)}$ containing 251 parameters (${\tilde{\bar{\beta }}}^{\left(L\right)}=\;\beta_{0}^{\left(L\right)},\beta_{1}^{\left(L\right)},\ldots ,\;\beta_{250}^{\left(L\right)}$). This large number of parameters renders the LH model a high-variance model, susceptible to large variations in coefficient estimates as a function of small changes to the training data set, and weakly generalizable to held-out data (38). To mitigate this statistical disadvantage while retaining the flexibility and mechanistic agnosticism we prize in this model, we minimize the NNLL in this model with an added L2 penalty,

(*4*) $${\tilde{\bar{\beta }}}^{\left(L\right)}=\underset{\tilde{\bar{\beta }}}{\text{arg min}}\left\{\text{NNLL}(\tilde{\bar{\beta }})+\text{ λ}{\text{‖}\tilde{\bar{\beta }}\text{‖}}_{2}^{2}\right\},\;{\text{‖}\tilde{\bar{\beta }}\text{‖}}_{2}^{2}=\;\sum_{p=\;0}^{250}{\tilde{\bar{\beta }}}_{p}^{2}\;$$

where the penalty term $\text{ λ}{\text{‖}\tilde{\bar{\beta }}\|}_{2}^{2}$ works to increase the NNLL. To minimize the NNLL in light of this penalty, the coefficient vector is optimized such that the most useful predictors are assigned large coefficients, while remaining predictors are assigned coefficients approaching zero, thus attenuating the effect of noise in parameter estimation. The regularization strength parameter, λ is set to equal 1 by default; we did no optimization over this parameter. LH model fits were performed using Python’s sklearn.linear_model.LogisticRegression method, and SH model fits ensured that the penalty argument was not active.

### Learning Phase Representations

The local field potential (LFP) is a complex signal, manifesting a number of oscillatory and nonoscillatory components at different times. Others have successfully leveraged the Fast Fourier transform of spike-triggered averages of the LFP as a predictor of synchronous spiking activity across multiple, simultaneously recorded units (39). Spike-triggered averages of the LFP may comprise multiple nested oscillatory and nonoscillatory components, and will thus represent a complex signal. In our work, we aim to tease apart the variety of oscillatory components present in the LFP, and use these to assess the timing of single-neuron spiking relative to the oscillatory process. Systematic spike timing relative to rhythmic phase may indicate a neuron’s engagement in the processes that give rise to the corresponding oscillatory LFP feature. Moreover, we wish to identify the potential reorganization of spike timing relative to the phase of different rhythms present in the LFP over time. For this reason, we have focused on modeling spike timing according to rhythmic phase.

Raw circular variables like oscillatory phase are inappropriate regressors in a generalized linear model (GLM). To generate a useful phase representation, we first noted that the spike-phase relationship can often be approximated by eye from a histogram of spike counts over phase. Leveraging this visual representation of the relationship, we applied kernel density estimation (KDE) by first repeating the distribution of spikes over phase to create three identical instances of the distribution and then convolving the three concatenated distributions with a Gaussian kernel. This procedure yields a smooth function representing the conditional probability of observing a phase ϕ given spiking (after dividing by three to account for the triple counting of spikes), p(ϕ |y=1) (Fig. 1*C*), which approximates the effect of convolving the data with a von Mises kernel.

The Gaussian kernel’s bandwidth (the width at half-maximum amplitude of the Gaussian kernel) crucially determines the ultimate shape of the kernel density estimator. To learn the optimal kernel bandwidth within a given interneuron data set, we applied fivefold cross validation on the training subset of trials [software package: Python sklearn, GridSearchCV, Kernel Density method, grid size = 1,000 (40)]. For the CA1 pyramidal cell datasets, we applied leave-one-out cross validation (instead of 5-fold cross validation) on the training subset of trials. This change ensured maximal use of the small amount of pyramidal cell spiking data available for kernel bandwidth selection.

On each fold of the cross-validation procedure, we sampled observations from the training set to generate a distribution of spiking over phase, and the optimal bandwidth (out of several possible bandwidths) was selected if its resulting estimator maximized the log likelihood over validation sets. It is important to explore the cross validated performance of a set of candidate kernel bandwidths that are sufficiently narrow or wide to capture fine or broad fluctuations of spiking as a function of phase. Increasing the total number of candidate kernel bandwidths will increase the computational time required to select the final bandwidth, but it will potentially result in a more accurate estimator. To estimate spike-phase relationships in this work, we explored the cross validated log likelihoods of 20 candidate bandwidths ranging from the equivalent of 6% to 40% of a phase cycle at kernel half-maximum. This range was selected to span a wide assortment of candidate bandwidths and smoothly capture slow or fast fluctuations of spiking over phase. This range is similar to the set of inverse variances deployed by Johnson et al. (41), who previously developed a successful method for estimating spiking relationships to oscillatory phase that differs substantively from the method currently applied here.

After estimating the probability of phases conditioned on spiking, we applied Bayes’ theorem to obtain the posterior probability of spiking given phase for the training set:

(*6*) $$\;p{\left(y=1|\;\phi \right)}_{\text{train}}=\;\frac{p{\left(\phi \text{|}y=1\right)}_{\text{train}}p{\left(y=1\right)}_{\text{train}}}{p(\phi )}$$

where the prior over spiking activity in the training set, *p*(*y* = 1)_train_, was computed as the average number of spikes divided by total time samples in the training set, and the prior over phase, *p*(ϕ), was uniform, $\frac{1}{2\text{π}}$. The probability of spiking given held out phase data, $\;p{\left(y=1|\;\phi \right)}_{\text{test}}$, was computed in the same way, but with likelihood $p{\left(\phi \text{|}y=1\right)}_{\text{test}}$, derived from the held-out distribution of spikes over phase. Importantly, we used the prior over spiking from the training set, to prevent the phase model from using any information in the held out set of spikes. These steps ensured that the held out set is a truly independent, and unbiased test of model performance.

### Models Combining Phase and History Information

We constructed models that account for both short and longer timescale temporal constraints by combining phase and history information. We fit logistic regression models to regressor combinations that included either short history and phase (“phaseSH models”), or long history and phase (“phaseLH models”):

(*7*) $$p\left(y_{i}=1\text{|}l_{i}^{\left(m\right)},l_{i}^{\phi }\right)=\;\frac{\exp\;(l_{i}^{\left(m\right)}{\text{β}}^{\left(lm\right)}+\;l_{i}^{\phi }{\text{β}}^{l\phi }+{\text{ β}}_{0}^{c(m)})}{1+\;\exp\;(l_{i}^{\left(m\right)}{\text{β}}^{\left(lm\right)}+\;l_{i}^{\phi }{\text{β}}^{l\phi }+{\text{ β}}_{0}^{c(m)})}.$$

In these models, we avoid re-fitting history coefficients by taking the log-odds of the probabilities over spiking generated by the long history, $l_{i}^{\left(L\right)}$, or short history, $l_{i}^{\left(S\right)}$. To include a phase representation that was suitable for logistic regression, we took the log-odds, $l_{i}^{\phi }$, of the $p\text{(}y_{i}=1\text{|}\phi_{i})$, for each time sample *i* derived from the training data. For models combining phase and history information, only three coefficients are fit: the intercept, ${\text{β}}_{0}^{c(m)}$ (with superscript *c*(*m*) to signal this coefficient’s applicability only to these combined models, as distinct from the history-only models specified in *Eq. 3*), and coefficients ${\text{β}}^{\left(lm\right)}$ and ${\text{β}}^{l\phi }$. Notably, these models will only fit these three parameters irrespective of the dimensionality of the originating history term, given that only the log-odds of the probability of spiking given history—and not the original history vector itself—is included in the models. The log-odds are expressed below,

(*8*) $$l_{i}^{\left(m\right)}=\;\log\;\frac{p(y_{i}=1|H_{i}^{\left(m\right)})}{1-\;p(y_{i}=1|H_{i}^{\left(m\right)})},$$

(*9*) $$l_{i}^{\phi }=\;\log\;\frac{p(y_{i}=1|\phi_{i})}{1-\;p(y_{i}=1|\phi_{i})}.$$

We then generated predictions over spiking using the held out data set with each of the models: short history only $p\left(y_{i}=1\text{|}H_{i}^{\left(S\right)}\right)$, long history only $p\left(y_{i}=1\text{|}H_{i}^{\left(L\right)}\right)$, phase $p(y_{i}=1|\;\phi_{i})$, phase and short history $p\left(y_{i}=1\text{|}l_{i}^{\left(S\right)},l_{i}^{\phi }\right)$, and phase and long history $p\left(y_{i}=1\text{|}l_{i}^{\left(L\right)},l_{i}^{\phi }\right)$. We refer to these models as SH, LH, phase, phaseSH, and phaseLH models, respectively, throughout the manuscript.

### Evaluating Model Performance and Goodness-of-Fit

To assess the likelihood-based goodness-of-fit (42) of models on the held out set of spikes, we computed the average log loss, LL, of each model,

(10) $$\text{LL}=\;-\frac{1}{n}\sum_{i=1}^{n}[y_{i}\;\log_{2}{\hat{y}}_{i}+(1-y_{i})\;\log_{2}\left(1-{\hat{y}}_{i}\right)],$$

where *i* corresponds to each 1 ms time sample, *y_i_* represents a spiking event in the held out data, 1 − *y_i_* represents the absence of a spiking event in the held out data, ${\hat{y}}_{i}$ corresponds to the predicted probability of a spike occurring according to the model, and 1 − ${\hat{y}}_{i}$ corresponds to the model’s predicted probability that a spike will not occur at time sample *i*. The log loss represents the degree of disparity between a model’s predicted spiking probability and the actual occurrence of a spike or its absence. As a consequence, smaller values indicate improved predictions.

### Identifying Neurons Exhibiting Spike-Phase Relationships

The phase model (as described in *Learning Phase Representations*) can fit a relatively flat line to spiking data that is uniform over all phases. This makes it difficult to assess whether the phase model performed well because it approximated the average probability of spiking or whether it performed well by approximating rhythmic structure. To identify cells with strong phase relationships, we computed the log loss (*Eq. 10*) of the phaseSH and SH models, and subtracted the log loss of the SH model from that of the phaseSH model. A negatively valued log loss difference indicates that including phase provides information beyond that encoded in the SH model (phaseSH log loss < SH log loss). In contrast, a log loss difference approaching zero indicates that phase is not adding much more information than the SH model contains by itself (phaseSH log loss ≈ SH log loss). We computed this log loss difference for each of the 20 train-test splits of data for each neuron, and then performed a one-tailed *t* test (*P* < 0.001) to assess whether the distribution of log loss differences was significantly smaller than zero, indicating that phaseSH models significantly outperformed SH models. This method effectively disambiguates neurons with systematic relationships to the phase of an LFP oscillation from those whose spiking is unrelated to the specified oscillatory phase.

### Characterizing Variations in Spike-Phase Relationships over Time

One substantial advantage of phaseSH models is their ability to make predictions over spiking at fine temporal resolution. To leverage this ability in individual neurons, we first fit phaseSH and SH models for each train-test split of the data in a behavioral epoch of interest, we bin the behavioral epoch into arbitrarily small window sizes (here, 50 ms, with no overlap), and then compute the mean log loss differences between the models for each train-test split of data (*n* = 20 unique log loss differences, one for each train-test split of data). This yields, for each 50 ms window, a distribution of log loss differences between the models we wish to compare (e.g., theta phaseSH to SH, or theta phaseSH to low gamma phaseSH, or low gamma phaseSH to SH). For each time window, we then used a two-tailed one-sample *t* test to assess whether the distribution of log loss differences (in that window) was significantly different from zero at a Bonferroni-corrected α of 0.00004 (that is, 0.001 divided by the total number of time windows, which corresponds to the number of *t* tests performed per neuron). The α was corrected to account for the multiple comparisons performed which, uncorrected, would dramatically inflate the likelihood of a Type I error.

### Rayleigh Test for Nonuniformity of Circular Distributions

To compare the results of the widely used Rayleigh test for nonuniformity against our model comparison approach to identify spike-phase relationships (described earlier), we computed the Rayleigh statistic for each neuronal spike train. The mean resultant length vector, *r*, is defined by,

(*11*) $$r\;=\;\sqrt{{\left(\frac{1}{\text{η}}\sum_{j\;=\;1}^{\text{η}}\text{cos}\left(\phi_{j}\right)\right)}^{2}+\;{\left(\frac{1}{\text{η}}\sum_{j\;=\;1}^{\text{η}}\text{sin}\left(\phi_{j}\right)\right)}^{2}},$$

where η corresponds to the total number of spikes in a spike train, and ϕ*_j_* represents a phase angle that co-occurred with spike *j*. The mean resultant length vector can then be used to compute the Rayleigh statistic, *Z*, where *Z* = *r*^2^/η.

Under this framework, the probability that a neuronal spike train generates a Z statistic of a particular magnitude given that the spike train was in fact drawn from a uniform distribution of spikes over phase is given by,

(*12*) $$p_{\text{Ray}}\;=\text{ exp}(\sqrt{1\;+\;4\text{η }+\;4({\text{η}}^{2}\;-\;R^{2})}\;-\;(1+2\text{η})),$$

where, as in *Eq. 11*, η corresponds to the total number of spikes in a given spike train, and *R* refers to the resultant length vector, expressed as *R* = η*r*.

### Code Availability

All code underlying the analysis methods described above is freely available at the following: https://doi.org/10.5281/zenodo.6837168.

## RESULTS

### Modeling Temporal Structure at Short and Long Timescales

To characterize the temporal structure of single-neuron spiking activity, we constructed five distinct models that exhibited sensitivity to spiking fluctuations at either short or long timescales, or a combination of the two. Here, short timescales refer to millisecond-resolution variations in spiking driven by afterhyperpolarization currents, whereas long timescales refer to a variety of potential mechanisms that could explain variations in spiking along tens to hundreds of milliseconds, including rhythmic synaptic currents. We applied these models to both simulated and electrophysiologically recorded datasets, with the recorded data drawn from previously published work that included CA1 pyramidal cells and inhibitory interneurons (35). This data set was recorded during an associative memory paradigm, where rats learned that odors in a pair were differentially rewarded depending on the context in which they were encountered. To receive a reward, rats maintained their position at the odor port containing the correct odor of a pair for 1,250 ms after delivery of the odor stimulus (see materials and methods). Our simulated data match the organizational structure of the CA1 recordings, with individual trials lasting 1,250 ms, and each trial preceded by an additional 250 ms of spiking history for the early time samples in the trial (Fig. 1*A*). For the present work, all model parameters (for both simulated and recorded datasets) were learned with a data set of training trials (50% of trials in the session), whereas the remaining trials were held out for performance evaluation.

We first modeled short timescale constraints on neuronal spike timing imposed by absolute and relative refractory periods. To do this, we constructed a logistic regression model whose predictions relied solely on the short history (3 ms) of the spike train (“SH model”, Fig. 1*B*). We additionally constructed a long history model (“LH model”, 250 ms) that captured longer timescale constraints on neuronal spiking activity, including (but not limited to) modulation by oscillatory currents. This 250 ms period corresponded to the period of a 4 Hz rhythm, ensuring that the LH model could capture temporal modulation of spiking activity at frequencies greater than or equal to 4 Hz (Fig. 1*B*). With 250 parameters to learn, we anticipated that LH models would be vulnerable to fitting noise in the training set. We nevertheless sacrificed predictive generalizability in this model to *1*) maximize the model’s ability to discover temporal structure at fine (and multiple) timescales, and *2*) make predictions with fine temporal resolution. For instance, any neuron in the data set that did not have a true relationship to the phase of the theta rhythm could still produce an LH model capable of capturing shorter timescale rhythmic fluctuations (e.g., low gamma, 35–55 Hz), neuronal adaptation dynamics, rebound spiking, as well as very fast membrane constraints (e.g., 1-ms refractory periods). Alternate models of long history have opted to bin temporal epochs or smooth spike times by applying various temporal filters, effectively averaging spike numbers over longer periods of time to reduce overfitting at the expense of temporal resolution (42). Windowing methods impose a set duration for relative refractory periods, which could vary widely across neurons, and also limit the fastest frequency modulation they can identify. Allowing millisecond-to-millisecond variation in our LH model increases the size of the family of functions we can fit to the data. To strike a balance between this model’s flexibility and its ability to generalize to new data (i.e., robustness), we imposed an L2 penalty on the LH model’s regression weights (43), which reduced noise in coefficient estimates while allowing the model to discover, at fine temporal resolution, any systematic relationships present in the data regardless of the underlying generative mechanism.

To specifically test the hypothesis that CA1 neuronal spike timing was systematically related to the phase of an ongoing oscillation, we first estimated the instantaneous phase of this oscillation at all time samples, and selected phase estimates that co-occurred with spiking events. We subsequently estimated the distribution of phases given a spike occurrence using a kernel density estimator with a Gaussian kernel whose bandwidth was optimized via fivefold cross validation, as described in material and methods. We then computed the probability of observing a spike *y_i_* = 1 given the instantaneous phase ϕ at every time sample *i*, or $p(y_{i}=\;1|\phi_{i})$, by applying Bayes’ transform to the kernel density estimator (Fig. 1*C*). Finally, to produce models that could capture temporal constraints on neural spiking activity at both short and long timescales, we constructed two logistic regression models that combined phase and history information: one containing phase and short history (“phaseSH models”), and another containing phase and long history (“phaseLH models,” Fig. 1*D*, see materials and methods).

### Model Performance on Simulated Data

**Figure 2.**
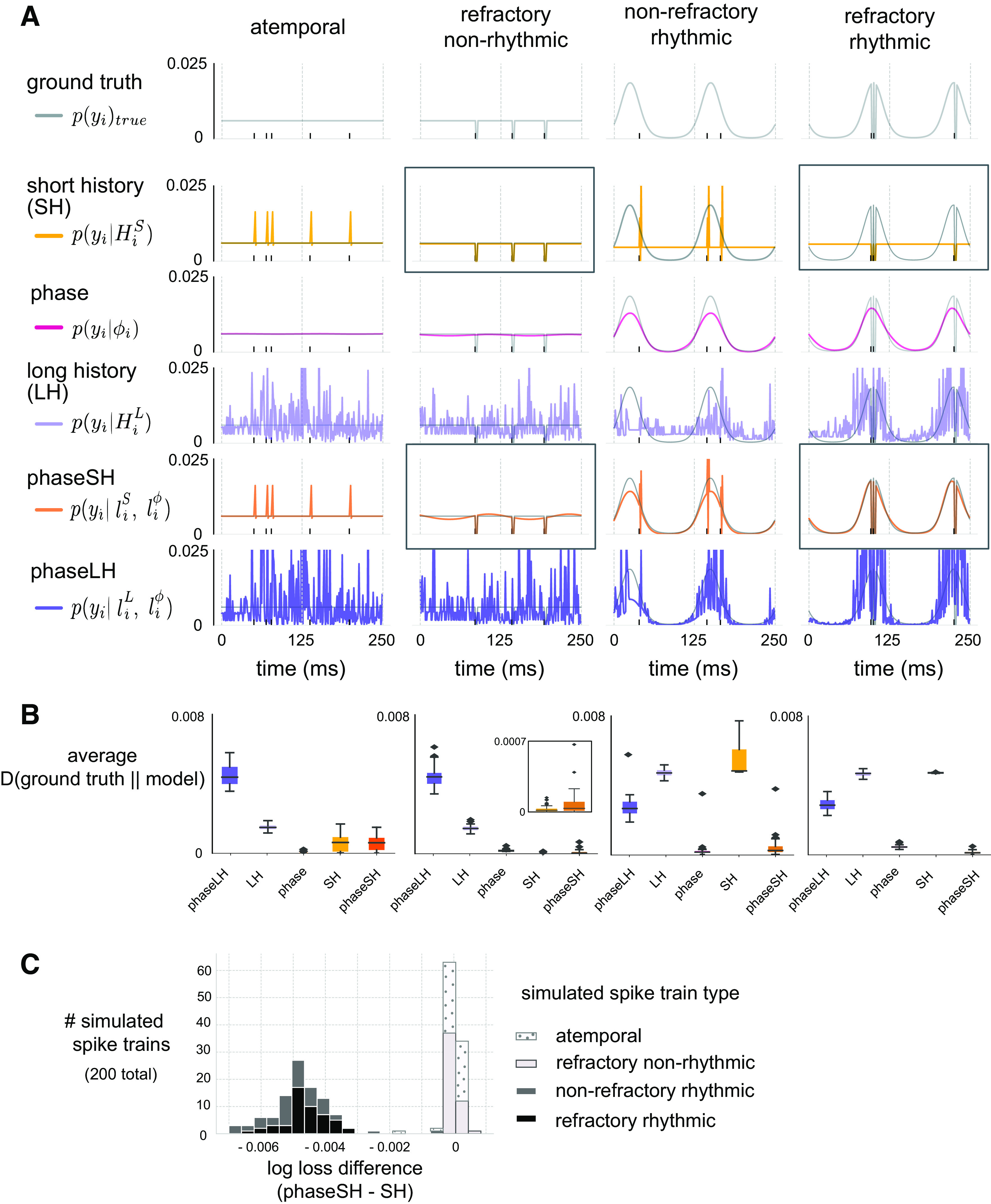
Model results in a simulated dataset. *A*: two cycles (250 ms) of held-out spiking (black ticks) for individual simulated spike trains and their corresponding ground truth probabilities (gray, all rows), or probabilities generated from short history (yellow, 2nd row), phase (pink, 3rd row), long history (light purple, 4th row), phaseSH (short history and phase, orange, 5th row), and phaseLH (long history and phase, dark purple, 6th row) models for four simulated spike trains corresponding to different “types,” where a “type” consists of a unique combination of rhythmic and refractory constraints. LH and phaseLH model heights are truncated to standardize axes across panels. Ground truth probabilities are replicated on each panel (within column) to facilitate visual comparisons across true and empirical model probabilities. Some model probabilities lie exactly along the ground truth for the entire time series (such as the phase model for the atemporal neuron). Vertical dashed lines mark the edges of two cycles of data. Boxed probability distributions highlight the qualitative performance of SH models and phaseSH models on neurons with either only refractory constraints, or both refractory and rhythmic constraints. *B*: average Kullback–Leibler (KL) divergences of the ground truth from a model’s probabilities across 50 simulated spike trains per type. Inset for the refractory nonrhythmic spike train type shows values more narrowly distributed along zero for SH models than for phaseSH models. Black horizontal bars mark the median, and whiskers mark 25th and 75th percentiles of the distribution. *C*: number of simulated spike trains (*n* = 200) by the difference in log losses between phaseSH and SH models.

We first validated the ability of each model to capture temporal structure at short and long timescales in simulated spiking data. We generated four “types” of spike trains, where spiking obeyed ground truth probabilities that incorporated a combination of short timescale (refractoriness) and/or long timescale (rhythmic modulation) constraints on spiking: termed hereafter atemporal, refractory nonrhythmic, nonrefractory rhythmic, and refractory rhythmic spike train types (Fig. 2*A*). A total of 50 simulated spike trains were created for each ground truth. The average target firing rate of these neuronal spike trains (6 Hz) approximated the average firing rate of a large proportion of CA1 inhibitory interneurons recorded during an associative memory paradigm (Supplemental Fig. S1; see https://doi.org/10.6084/m9.figshare.16663216).

Each model produced systematic predictions (and deviations from the ground truth) that varied according to simulated spike train type (Fig. 2*A*). To quantify deviations between model predictions and the ground truth of each type of spike train, we first leveraged an information-theoretic approach. This consisted of computing the average Kullback–Leibler (KL) divergence between the ground truth spiking probabilities and the empirically derived, model-based probabilities (see *Data Simulations*). Small (∼0) KL divergences between the ground truth and model-based probabilities indicated little to no deviation from the ground truth, and thus demonstrated an ability to fit the temporal structure in the spike train. Larger KL divergences, on the other hand, indicated systematic deviations from the ground truth and demonstrated an inability to capture essential sources of variance in the spike train.

SH models incurred the smallest and least variable KL divergences for simulated spike trains exhibiting a refractory constraint but no phase modulation (refractory nonrhythmic spike trains), and produced systematically large KL divergences from the ground truth whenever spiking was rhythmically modulated (nonrefractory rhythmic and refractory rhythmic; Fig. 2*B*, *middle* and *far right*). In cases where simulated spike trains did not have a refractory period, SH models predicted increased firing within milliseconds following a spike in the history (atemporal and nonrefractory rhythmic; Fig. 2*A*, second row, *far left* and *middle right*). SH models thus excelled at capturing variance in spike trains containing refractory constraints but no rhythmic phase modulation and, as expected, dramatically failed to capture variance in spike trains modulated by rhythmic phase.

Phase model predictions flexibly adapted to each simulated spike train’s ground truth, capturing rhythmic phase modulation when it existed (nonrefractory rhythmic, and refractory rhythmic), and basing predictions on the average spike rate when it did not exist (atemporal, and refractory nonrhythmic). These properties allowed the phase model to cleanly approximate the ground truth probabilities in the atemporal and nonrefractory rhythmic spike trains, with very subtle deviations from the flat ground truth in the former case. The phase model incurred slightly larger KL divergences from the ground truth whenever the latter incorporated a refractory constraint (refractory nonrhythmic, and refractory rhythmic), but these KL divergences were relatively infrequent given the low firing rate of these simulated spike trains (Fig. 2*B*). At faster spike rates, however, phase models dramatically diverged from the ground truth for spike trains governed by refractory constraints (Supplemental Fig. S2*A*, *middle left* and *far right*; see https://doi.org/10.6084/m9.figshare.20293863). Phase models were thus adept at capturing variance in rhythmically modulated spike trains, but suffered from their inability to account for afterhyperpolarizations.

LH models were susceptible to spurious patterns in data whose ground truth was either completely uniform (atemporal) or only contained refractory constraints (refractory nonrhythmic), despite reducing noisier coefficient estimates through L2-regularization. These models consequently suffered KL divergences from the ground truth that were relatively larger than those incurred by phase and SH models for the nonrhythmic simulated spike trains (Fig. 2*B*, *far* and *middle left*). This model was, however, able to capture rhythmic temporal structure in spike trains whose ground truths incorporated phase modulation, albeit noisily (nonrefractory rhythmic, refractory rhythmic; Fig. 2*A*). LH models, as formulated here, were not ideal for capturing rhythmic constraints on spiking.

PhaseSH models closely matched SH model predictions for simulated spike trains that had no phase modulation, performing slightly worse than SH model predictions (atemporal, and refractory nonrhythmic; Fig. 2*B*, *far* and *middle left*). This was likely a consequence of including the phase component which, while close to uniform, was not quite flat (Fig. 2*A*, *left boxed panel, fifth row of models*). As with the SH models, in cases where there was no refractory constraint, the models tended to overestimate the probability of spiking immediately following a spike (atemporal, and nonrefractory rhythmic; Fig. 2*B*, *far left*, *middle right*). PhaseSH models best approximated the ground truth probabilities for spike trains that exhibited both refractory and rhythmic constraints (refractory rhythmic; Fig. 2*B*, *far right*). PhaseSH models were thus well suited for capturing rhythmic constraints when they existed, readily captured variation in spike trains for simulated spike trains with both slow (∼6 Hz) and fast (∼40–60 Hz) average firing rates (Supplemental Fig. S2*A*), and approximated SH model predictions when rhythmic constraints were not present in the data.

Finally, phaseLH model predictions behaved similarly to LH models, capturing spurious patterns in atemporal and refractory non-rhythmic spike trains (Fig. 2*B*, *far* and *middle left*), but could approximate oscillatory spike distributions over time due to the combined contributions of LH and phase models (nonrefractory rhythmic, and refractory rhythmic; Fig. 2*A*, sixth row). Noisy estimates, however, resulted in large and frequent KL divergences from the ground truth (Fig. 2*B*). In simulations, phaseLH models were thus less adept than phaseSH models at accurately describing rhythmic constraints in spike trains.

Using the phaseSH models, we hoped to identify neurons whose spike trains were specifically influenced by rhythmic currents and were thus systematically related to the phase of an LFP oscillation. Given the phase model’s ability to produce reasonably smooth and accurate predictions even in cases where simulated spike trains did not exhibit phase modulation, we were not able to use phase model performance alone to identify the neurons we sought. Instead, we first noted that disparities in predictions between the phaseSH model and the SH model should lead to differences in likelihood-based measures of goodness-of-fit (42). We consequently took a model comparison approach and computed the log losses of phaseSH and SH models (evaluated on held out trial data, see *Evaluating Model Performance and Goodness-of-Fit*). We then subtracted these log losses to produce the log loss difference between models (see *Identifying Neurons Exhibiting Spike-Phase Relationships*). Log loss differences closer to zero resulted from similar performance between phaseSH and SH models, indicating that oscillatory phase failed to provide information above and beyond what was already encoded in SH model predictions. This was precisely the pattern observed for atemporal and refractory nonrhythmic simulated spike trains, whose temporal structure was unrelated to oscillatory phase (Fig. 2*C*). In contrast, log loss differences much smaller than zero emerged when the phaseSH model’s predictions held an advantage over SH model predictions, indicating that oscillatory phase information improved the likelihood-based goodness-of-fit. A similar pattern of results emerged when using KL divergences to compare phaseSH and SH models (Supplemental Fig. S3; see https://doi.org/10.6084/m9.figshare.16663231). Notably, this was exactly the result obtained for nonrefractory rhythmic and refractory rhythmic simulated spike trains, which were systematically related to oscillatory phase (Fig. 2*C*; Supplemental Fig. S2*B*). Using likelihood-based model comparisons between the phaseSH and SH models, we were able to successfully recover the simulated spike trains whose spike probability was organized according to oscillatory phase.

### Model Application to CA1 Neurons

We subsequently applied all models to CA1 inhibitory interneurons, where we aimed to identify interneurons in the data set whose spike timing preferences were systematically related to the phase of the theta (4–12 Hz) rhythm. We additionally replicated this analysis using the phase of the low gamma (35–55 Hz) rhythm. For each rhythm separately, we trained each of the five models for every interneuron in the data set, and subsequently designed a series of model comparisons to determine *1*) whether phase-based models could more accurately predict spike timing in unseen trials relative to long history-based models, and *2*) whether interneuron spiking could be reliably explained specifically by rhythmic currents, as measured from the LFP, across trials.

#### Comparing phase-based and long history-based model performance.

**Figure 3.**
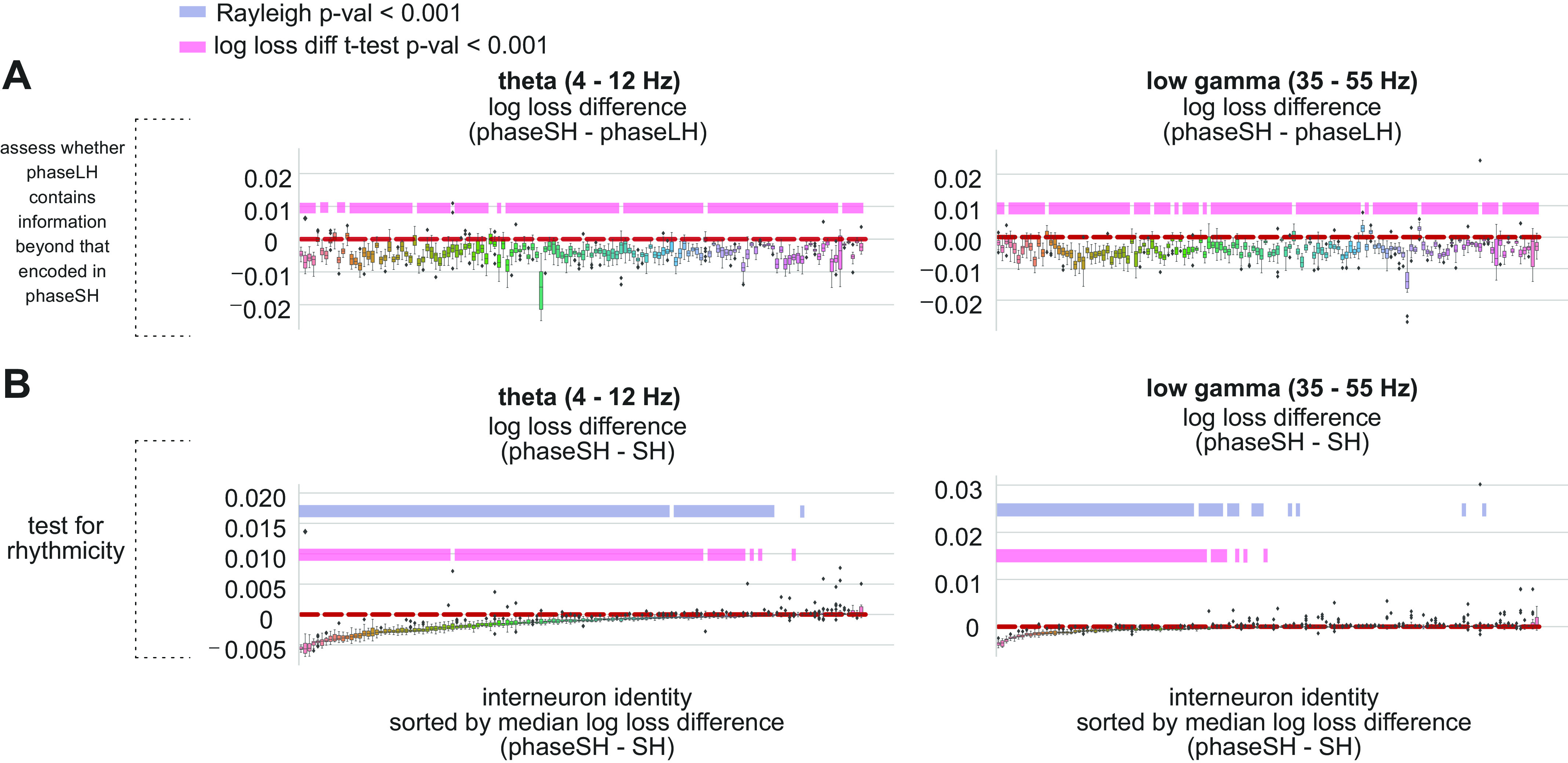
Model comparisons for odor sampling CA1 inhibitory interneuron data, for theta- and low gamma-phase based models separately. *A*: comparisons between models combining theta phase and short history (phaseSH) and models combining theta phase and long history (phaseLH) (*left*) and low gamma phaseSH and low gamma phaseLH models (*right*). Differences in the corresponding model log losses (*y*-axis) evaluated on held-out data, across 20 folds (train-test splits) of data for each CA1 interneuron spike train (*x*-axis). Black horizontal bar within boxplots marks the median, whiskers mark the 25th and 75th percentiles of the distribution of folds, diamonds mark outliers. A log loss difference of zero (marked by dotted red line) indicates equal performance across phaseSH and phaseLH models. Pink horizontal bars mark the neuronal spike trains whose log loss difference distributions were significantly smaller than zero (*P* < 0.001), according to a one-tailed *t* test. Interneuron spike train identities are sorted according to the median of the log loss difference between phaseSH and SH models across folds. *B*: model comparisons between theta phaseSH and SH models (*left*), and low gamma phaseSH and SH models (*right*). All visualization features remain the same as in *A*, save the specific models being compared. Gray bars mark interneuron spike trains whose spike-phase distributions (drawn from the combined train and test trial data) significantly differed from uniform according to a Rayleigh test for nonuniformity (*P* value <0.001). Pink horizontal bars mark the neuronal spike trains whose log loss difference distributions were significantly smaller than zero (*P* < 0.001), according to a one-tailed *t* test.

To address this first question, we quantified the proportion of interneurons in this data set whose phaseSH model reliably outperformed the phaseLH model. To test for reliability, we subtracted the phaseLH model log loss from the phaseSH model log loss for each of the 20 train-test splits of data, yielding a distribution of 20 log loss differences for each neuronal spike train. If, for a given spike train, the phaseSH model smoothly recapitulated all the relevant information embedded in the much more detailed but mechanism-agnostic phaseLH model, then we expected the distribution of log loss differences across the 20 folds to be significantly smaller than zero according to a one-tailed *t* test, indicating that long history-based models overfit to the training set and were unable to generalize to held out data from the same neuronal spike train. With few notable exceptions, we found that the phaseSH models based on theta (Fig. 3*A*, *left*) and low gamma (Fig. 3*A*, *right*) phase information were able to account for variation in held-out interneuron spike trains more readily than phaseLH models during the odor sampling epoch (theta: 91% interneuron spike trains, *n* = 122 of 134, one-tailed *t* test, *P* < 0.001; low gamma: ∼89% interneuron spike trains, *n* = 120 of 134, one-tailed *t* test, *P* < 0.001). This trend was reversed when log losses were evaluated on the training trial data (Supplemental Fig. S4; see https://doi.org/10.6084/m9.figshare.16663240), where the phaseLH models held a clear advantage (unsurprisingly) over the phaseSH models.

#### Identifying neurons with reliable rhythmic engagement.

To address the second question, we aimed to assess how reliably rhythmic currents in the LFP accounted for variance in neuronal spike trains. To achieve this, it was necessary to compare model performance between phaseSH and SH models, a model comparison that we previously established as a suitable metric to assess reliable spike-phase relationships to rhythmic currents measured from the LFP (Fig. 2*C*; Supplemental Fig. S2*B*). We compared the log losses for these models for the same 20 train-test splits used previously (Fig. 3*A*), and compared the distribution of log loss differences for each interneuron spike train against the null hypothesis that there was either no difference across the model log losses (which would indicate no effect of rhythmic currents on spike train temporal structure) or that this difference was larger than zero (which would indicate that the SH model outperformed the phaseSH model). This metric resulted in ∼80% (*n* = 107 of 134, one-tailed *t* test, *P* < 0.001) of interneuron spike trains exhibiting log loss differences that were reliably smaller than zero, suggesting that theta phase robustly explained variance in these neurons’ spike trains (Fig. 3*B*, *left*). We also performed this analysis for phaseSH models leveraging low gamma phase information and found that 44% of interneuron spike trains (*n* = 59 of 134, one-tailed *t* test, *P* < 0.001) exhibited log loss differences that were reliably smaller than zero (Fig. 3*B*, *right*).

We additionally compared the results of the log loss differences between phaseSH and SH models against the results of a Rayleigh test for nonuniformity in circular distributions (Fig. 3*B*, *bottom gray bars*). Few discrepancies between the metrics emerged when identifying neuronal relationships to the theta rhythm, amounting to ∼8% of interneuron spike trains (*n* = 11 of 134; where the Rayleigh test identified 9 of 11 neurons missed by the model comparison approach, and the latter identified 2 of 11 neurons missed by the Rayleigh test; Fig. 3*B*, *left*). Discrepancies between the metrics also arose when assessing low gamma relationships. Here, the disparities amounted to ∼10% of interneuron spike trains (*n* = 14 of 134; Rayleigh test: identified 10 of 14; model comparisons: identified 4 of 14; Fig. 3*B*, *right*). The exceptions highlight a crucial difference between the Rayleigh test and the phaseSH model. The Rayleigh statistic exclusively accounts for number of spikes pooled across all trials, irrespective of the total number of time samples available for the recording. The phaseSH model, on the other hand, accounts for firing rates (the number of spikes and the number of total time samples), and our cross-validation approach implicitly assesses trial to trial reliability of spiking. With our modeling approach, a cell is identified as significantly entrained to a particular rhythm if its baseline firing rate is robust and its spike timing is reliably reproducible across trials. The Rayleigh statistic and phaseSH models can thus offer complementary information: the Rayleigh statistic reveals whether a cell emits spikes biased toward particular phase angles, and phaseSH models assess the predictability and reliability of spikes occurring at particular phase angles.

This effect was particularly evident when we tested model performance on CA1 pyramidal cell data. Although CA1 pyramidal cells in this data set expressed high firing rates during odor sampling intervals for particular positions, odors, and odor position combinations (35), there were only six trials available for every odor position combination. Given the high degree of selectivity for certain stimulus combinations, we analyzed pyramidal cell spike trains for each odor position combination separately, yielding only six trials per pyramidal cell spike train: a much smaller number of available trials for training and testing than even some of the sparsely firing inhibitory interneurons (Supplemental Fig. S6; see https://doi.org/10.6084/m9.figshare.19617693). This resulted in larger discrepancies between the Rayleigh statistic and theta phase-based model comparisons, particularly when attempting to identify neurons whose spiking was related to the theta rhythm. While the Rayleigh statistic identified 35% of pyramidal cell spike trains as related to the theta rhythm (*n* = 15 of 43 pyramidal cell spike trains, *P* < 0.001), the model comparison approach identified 12% of pyramidal cell spike trains as reliably related to the theta rhythm (*n* = 5 of 43, one-tailed *t* test, *P* < 0.001). These discrepancies were dramatically reduced when identifying neurons whose spiking was related to the low gamma rhythm. Here, the Rayleigh statistic and the low gamma phase-based model comparisons yielded closer agreement, where the Rayleigh statistic identified ∼7% of pyramidal cell spike trains as related to low gamma (*n* = 3 of 43, *P* < 0.001), while the model comparison approach identified ∼5% of pyramidal cell spike trains as reliably related to low gamma (*n* = 2 of 43, one-tailed *t* test, *P* < 0.001). In each case, the phase-based model comparison approach tended to yield more conservative estimates of spike-LFP relationships than those produced by the Rayleigh statistic, given the phaseSH model’s ability to account for both the probability of spiking over time as well as the rhythmic temporal structure of spiking.

To further verify that population-level metrics presented in Fig. 3 were accurately selective for rhythmic temporal structure in spike trains, we closely evaluated models and their performance on a subset of sample CA1 interneuron spike trains strategically drawn from the population to represent the median log loss difference at the 0th (most negative median log loss difference, marking the most predictive theta phaseSH model), 25th, 49th, 51st, 75th, and 100th percentiles of the distribution of all CA1 interneuron median log loss differences (Supplemental Fig. S7; see https://doi.org/10.6084/m9.figshare.19621851). This selection procedure ensured close inspection of a wide range of neuronal spike trains, some of which should be reliably leveraging phase information specifically to achieve accurate phaseSH model predictions above and beyond the average firing probabilities represented by their SH models. With this subset of neurons, we specifically asked: how much did phase information contribute to the performance of each of the models that included it? To address this question, we trained a hundred versions of each model whose predictions were either partially or completely based on phase data (phase, phaseSH, and phaseLH models), with phase values shuffled randomly within a single cycle while keeping spike history intact. We then computed the log losses evaluated on the held out data for the phase-permuted models, as well as those from the unperturbed models.

For interneuron spike trains drawn from the 0th to the 51st percentile of the distribution of median log loss differences, it was clear that models using intact phase information produced log loss values that were far smaller than the most extreme values of the distribution of log losses from the models trained on phase-permuted data (Supplemental Fig. S7). The log loss values for LH and SH models are included for reference. This predictive accuracy metric supported the results from the population metrics in Fig. 3*B*, suggesting that interneuron spike trains whose log loss differences between phaseSH and SH models are significantly smaller than zero have spike trains whose temporal structure is reliably related to the phase of the theta rhythm.

#### Characterizing rhythmic engagement profiles across behavioral epochs.

**Figure 4.**
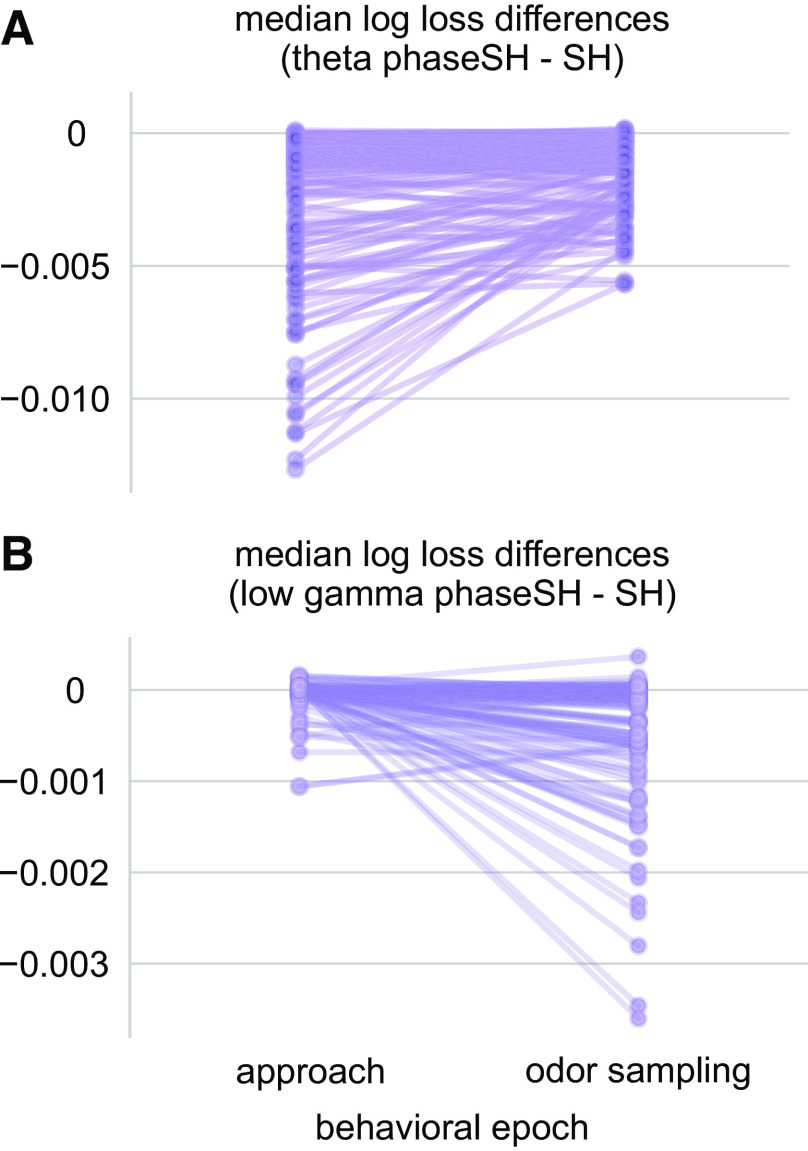
Models combining phase and short history (phaseSH) capture shifts in CA1 interneuron spike-local field potential (LFP) relationships across distinct behavioral epochs. *A*: each line represents the median log loss difference between the theta phaseSH model and the SH model trained on a single interneuron’s spike train in either the running approach or odor sampling epoch. A difference in height between the approach and odor sampling behavioral epochs thus indicates a change in a spike train’s degree and reliability of entrainment to the theta rhythm across trials. *B*: Same as in *A*, for the low gamma phaseSH models. Note the scale differences across *A* and *B*. More negative values indicate that the phaseSH model outperformed the SH model.

The temporal structure of neuronal spiking varies across distinct behaviors (2–4). Here, we additionally apply model comparisons between phaseSH and SH models to evaluate hypothesized rhythmic current influences across different behavioral epochs during the associative memory task. We specifically aimed to visualize shifts in rhythmic current influences across the running approach to the odor port and the subsequent odor sampling epoch when the CA1 region was likely engaging associative memory processes. The pattern of model comparisons reveals a qualitative shift between the approach and odor sampling epochs (explored quantitatively in Supplemental Fig. S5; see https://doi.org/10.6084/m9.figshare.19684068). Although interneuron spike trains appear to be strongly related to rhythmic currents in the theta frequency range, this degree of relationship tends to shrink during the odor sampling epoch (Fig. 4*A*). On the other hand, relationships to low gamma-range rhythmic currents tend to increase during the odor sampling epoch relative to their influence during the approach epoch (Fig. 4*B*). Model comparisons between phaseSH and SH models can reveal differences in the degree of influence that rhythmic currents exert across distinct behavioral epochs.

#### Characterizing rhythmic engagement profiles that change over time.

We next sought to leverage the temporal resolution of the phaseSH models to characterize the time course of single neuron relationships to rhythmic processes, such as the theta (4–12 Hz) and low gamma (35–55 Hz) rhythms, as reflected in the local field potential (LFP). In the hippocampus, running epochs coincide with the dominant presence of theta oscillations (44, 45), and running speed is positively correlated with increasing interneuron firing rates (14, 17). Accordingly, we hypothesized that behavioral epochs during which rats were running toward odor ports (approach epochs) would likely coincide with high rates of CA1 interneuron spike phase relationships to the theta rhythm relatively consistently throughout the approach interval. In contrast, the odor sampling epoch of the associative memory task requires parsing multiple input streams to integrate the relevant contextual and odor information that should guide the rat’s successful behavior. We thus hypothesized that some interneurons would be more likely to exhibit shifts in spike phase relationships to distinct CA1 rhythms during this odor sampling, associative memory window.

To characterize the time course of spike-phase relationships during the approach and odor sampling epochs, we again compute differences in model log losses on held-out data to ask whether the relative predictive accuracies between the theta and low gamma phaseSH models varied over the course of associative memory processing within the odor sampling epoch, for each interneuron independently. The millisecond resolution of phaseSH models allowed us to track whether any given held-out spike was better predicted by the probabilities generated from the theta or low gamma phaseSH models. We could then aggregate these log losses within arbitrarily small or wide window sizes (in this case 50 ms duration with no overlap) and take the median of these across results from the 20 train test splits of the data within the odor sampling window to obtain an estimate of the relative strength of the relationship to either of the two rhythms.

We present the median log loss differences (computed over time) between the following models: theta phaseSH minus SH, theta phaseSH minus low gamma phaseSH, and low gamma phaseSH minus SH. Across the population, this analysis captured the relatively weaker low gamma rhythmic current influences on spiking during the approach epoch (Supplemental Fig. S8, *A*–*C*, *left*; see https://doi.org/10.6084/m9.figshare.19678359; Fig. 4, Supplemental Fig. S5). During the odor sampling epoch, however, transient periods emerged during which low gamma phaseSH models outperformed theta phaseSH models (Supplemental Fig. S8*B*, *right*). This pattern of low gamma rhythmic current influence bore out when comparing the theta phaseSH model against the SH model: along the diagonal of the matrix, the accuracy of the theta phaseSH model tended to suffer (Supplemental Fig. S8*A*, *right*), whereas model comparisons between the low gamma phaseSH and SH models revealed slight increases in the influence of low gamma rhythmic currents on spike timing (Supplemental Fig. S8*C*, *right*).

**Figure 5.**
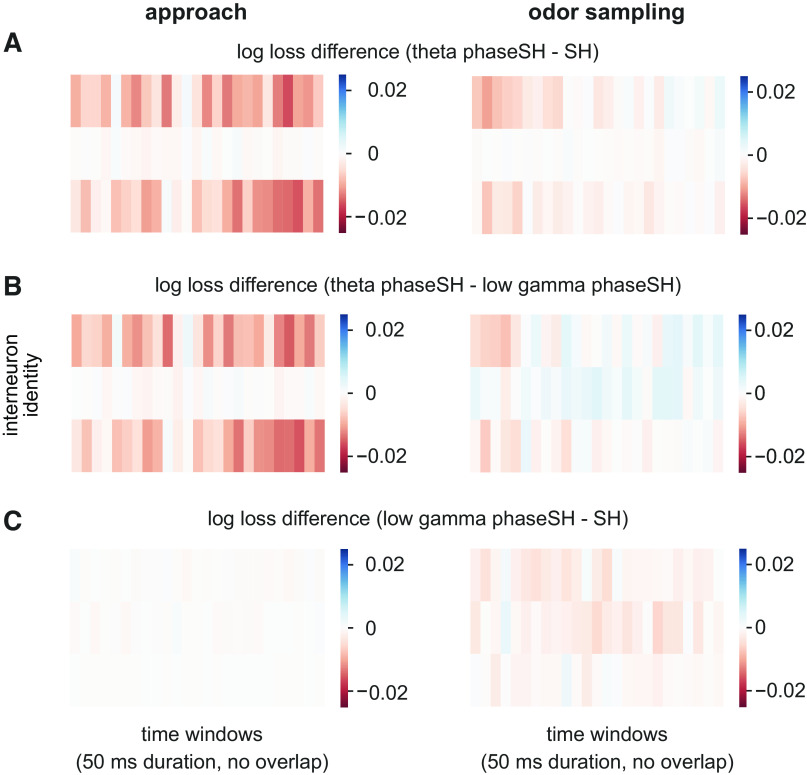
Model comparisons over time reveal rapid shifts in spike-phase relationships to local field potential (LFP) rhythms during an odor sampling epoch. Models were fit to spiking and rhythmic phase data from behavioral epochs when rats were either running toward the odor port (*left column*, approach) or odor sampling (*right column*). *A*: rows correspond to three unique interneuron identities, matched across approach and odor sampling behavioral epochs. Columns within each heatmap correspond to nonoverlapping 50-ms long time windows spanning a continuous interval of 1,250 ms. Color scale corresponds to log loss differences between models combining theta phase and short history (phaseSH) and SH models computed on held-out data for each of the 20 train test splits. Warmer red colors indicated that theta phaseSH models outperformed SH models. The average firing rates across all 20 held out sets during the approach are as follows, for each neuron in order of rows from top to bottom: 35.5 Hz, 36.6 Hz, 42.4 Hz. For the odor sampling epoch, the average firing rates are as follows: 20.3 Hz, 20.9 Hz, 26.2 Hz. *B*: same as in *A*, for the same three neurons, but for log loss differences between the theta phaseSH model and the low gamma phaseSH model. Warmer red colors indicated better performance by theta phaseSH models, while cooler blue colors indicated better performance by low gamma phaseSH models. *C*: same as in *A*, but for log loss differences between low gamma phaseSH models and SH models. Warmer red colors indicated better performance for low gamma phaseSH models. All color maps are displayed on the same scale.

**Figure 6.**
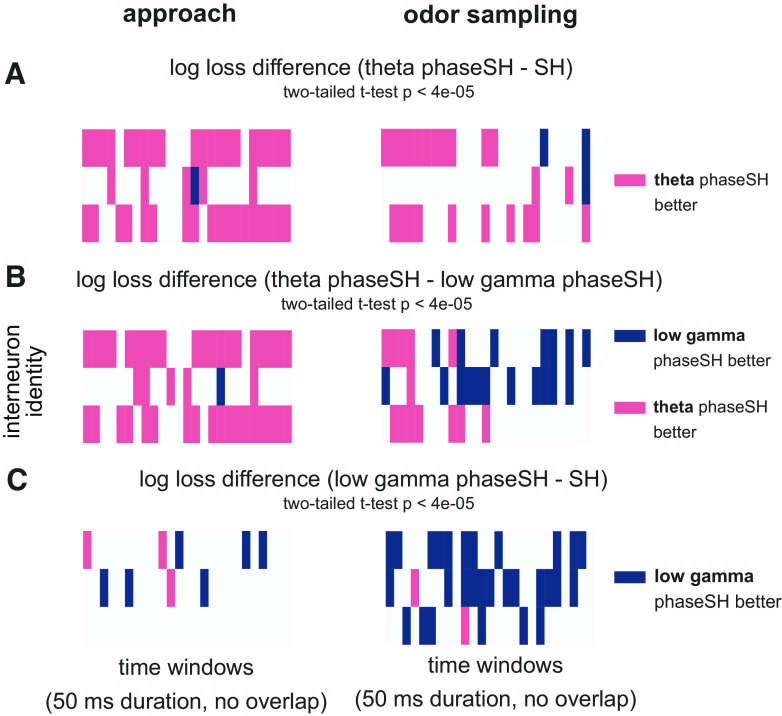
Model comparisons over time on held-out data, with significance assessed per time window using a two-tailed one-sample *t* test. As in Fig. 5, models were fit to spiking and rhythmic phase data from behavioral epochs when rats were either running toward the odor port (*left column*, approach) or odor sampling (*right column*). Model performance was tested on the held-out trials of each train-test split. *A*: rows correspond to three unique interneuron identities, matched across approach and odor sampling behavioral epochs. Columns within each heatmap correspond to nonoverlapping 50-ms long time windows spanning a continuous interval of 1,250 ms. Colors correspond to log loss differences between models combining theta phase and short history (phaseSH) and SH model computed on held-out data for each of the 20 train test splits that were significantly different from a distribution centered around zero according to a two-tailed one-sample *t* test (*n* = 20 log loss differences per window, *P* < 0.00004). Pink bars in heatmaps correspond to time windows whose distribution of log loss differences indicate better theta phaseSH model predictions relative to SH model predictions; blue indicates SH model predictions significantly outperformed theta phaseSH model predictions. *B*: same as in *A*, for the same three neurons, but for significant log loss differences between the theta phaseSH model and the low gamma phaseSH model. Pink in these heatmaps indicates that the theta phaseSH model significantly outperformed the low gamma phaseSH model; blue indicates that low gamma phaseSH models significantly outperformed the theta phaseSH models. *C*: same as in *A*, but for significant log loss differences between low gamma phaseSH models and SH models. Blue in these heatmaps indicates low gamma phaseSH models significantly outperformed SH models; pink indicates that SH models outperformed low gamma phaseSH models. White bars indicate no significant difference in model performance.

To further explore the range of trends in rhythmic influence, we selected a subset of neuronal spike trains with dynamic outcomes in the model comparisons over time. Notably, one of the three sample interneuron spike trains exhibited relatively few time windows with significant theta or low gamma rhythmic current influences during the approach or the odor sampling epoch (Fig. 5, *A–C* and Fig. 6, *A*–*C*, *left*, *middle rows*, two-tailed one-sample *t* test, *P* < 0.00004, *n* = 20 log loss differences per time window), but it did exhibit what appeared to be a relatively sustained relationship to low gamma rhythmic currents during the odor sampling epoch (Fig. 5*B* and Fig. 6*B*, *right, middle rows*, two-tailed one-sample *t* test, *P* < 0.00004, *n* = 20 log loss differences per time window). The other two sample interneuron spike trains exhibited relatively strong and consistent theta relationships during the approach (Fig. 5, *A* and *B*, Fig. 6, *A* and *B*, *left, top* and *bottom rows*, two-tailed one-sample *t* test, *P* < 0.00004, *n* = 20 log loss differences per time window), and weaker low gamma relationships during this interval (Fig. 5*C* and Fig. 6*C*, *left*, two-tailed one-sample *t* test, *P* < 0.00004, *n* = 20 log loss differences per time window). During the odor sampling epoch, however, the theta phaseSH models for these neurons decreased in predictive utility over the course of the odor sampling epoch (Fig. 5, *A* and *B* and Fig. 6, *A* and *B*, *right*, *top* and *bottom rows*, two-tailed one-sample *t* test, *P* < 0.00004, *n* = 20 log loss differences per time window). Surprisingly, when the theta rhythmic influence decayed in one of these spike trains, the low gamma rhythmic influence acquired prominence toward the end of the epoch (Fig. 5*B* and Fig. 6*B*, *right, top row*, two-tailed one-sample *t* test, *P* < 0.00004, *n* = 20 log loss differences per time window).

Model comparisons evaluated over time can thus test the hypothesis that specific rhythmic currents shape spike timing at fine time resolution, and the phaseSH model’s failures and successes in predictive utility have the potential to reveal rapid shifts in spike timing.

## DISCUSSION

Neuronal spike timing and its rapid dynamics equip organisms to successfully adjust to a shifting landscape of sensory information and behavioral demands. With the present work, we offer a modeling approach that can *1*) capture short-lived intrinsic constraints on spiking as well as rhythmic current influences that unfold over relatively longer timescales, and *2*) evaluate neuronal recruitment by a constellation of distinct rhythmic currents, as measured from the local field potential (LFP), over time. Specifically, models including the immediate spiking history and information about the phase of an LFP oscillation—termed “phaseSH” models in this work—can recover short and rhythmic long timescale constraints on cellular spiking. Importantly, we prescribe approaches to identify neurons in large datasets that exhibit systematic relationships to LFP rhythms. By innovatively applying long-established statistical modeling methods and their associated performance metrics, these models can make informed (and verifiable) predictions over spiking at fine time resolution, rather than collapsing estimates across long windows of time and assuming consistent temporal structure during these periods. With our statistical models, it is possible to explicitly test neuronal participation in rhythmic processes at exquisite temporal resolution.

In this work, we propose a novel application of kernel density estimation (KDE) to estimate the conditional probability of spiking given the phase of an LFP oscillation and we apply these predictions over time. To specifically test the hypothesis that spike timing is related to the phase of an oscillation (and is thus likely influenced by rhythmic currents), it is imperative to construct models that avoid imposing distributional biases (e.g., normality) on their relationship. Biased models may fail to recover various features (e.g., variance and complexity) of the true underlying relationship and result in hypothesis tests using unrepresentative relationships. With KDE, we can instead empirically estimate the relationship from training data. We first estimate the likelihood of theta (or low gamma) phases given spike occurrences using KDE (Fig. 1*C*). We subsequently compute the posterior probability of observing a spike given observed phase values, and we apply this probability mapping over the duration of the phase timeseries to produce probabilities of spiking that unfold over time (Fig. 1*C*). Crucially, we leverage cross validation to select the optimal kernel bandwidth used to convolve the distribution of phase observations that co-occurred with spikes. Our use of KDE to estimate the conditional probability of spiking given phase, and our ability to produce an optimized estimator, presents an innovative contribution to the problem of characterizing spike-phase relationships.

Our goal was to apply KDE-based “phase” model predictions over time. Phase model predictions suffered, however, from the inability to account for short timescale refractory constraints (Fig. 2*B*, Supplemental Fig. S2*A*). For this reason, we additionally constructed logistic regression models using the 3 ms short (“SH”) history of spiking, and combined the predictions from this model with those from the phase model (Fig. 1*D*). This combination yielded the “phaseSH” model, which was capable of adjusting predictions according to refractory constraints while simultaneously testing whether particular rhythmic currents explained variance in spike trains (Fig. 2). PhaseSH models captured ground truth rhythmic dynamics in simulated spike trains and they did so more accurately than models whose predictions were based on the 250 ms long (“LH”) history of spiking (Fig. 2*B*).

Simulations also revealed a key property of phaseSH models: they produced flat predictions over time whenever spikes were not systematically organized according to the phase time series being tested. This property allowed us to leverage model comparisons between phaseSH and SH models to identify simulated (Fig. 2*C*; Supplemental Fig. S2*B*) and CA1 neuronal spike trains (Fig. 3*B*; Supplemental Figs. S5 and S6) that exhibited reliable rhythmic relationships.

Alternative statistical approaches to quantifying rhythmic temporal structure in spike trains exist. Of these, the Rayleigh statistic and mean resultant length vectors are prominently used in the field (35, 46). Although a useful metric to distinguish the density of spiking concentrated along some phases (example in Fig. 1*C*) from a uniform distribution, the Rayleigh statistic requires pooling spikes over large windows of time. In contrast, our cross-validated model comparison approach allowed us to quantify the predictive utility of phaseSH models on held out data, enabling assessment of its predictions both across trials (Fig. 3) and within trials over arbitrarily small time samples down to millisecond resolution (Fig. 4). These features allow us to examine the trial-to-trial reliability of spike-phase relationships, as well as identify rapid shifts in spike-phase relationships over the course of behavior. A strength of the Rayleigh statistic, however, is that it will not take baseline firing rate into account, unlike our KDE and logistic regression models. This means that its estimates of spike-phase relationships will not distinguish between neurons exhibiting vastly different firing rate behavior, as long as each neuron provides the same total number of spikes. A sparsely firing neuron recorded over a long session may produce the same number of spikes, and obtain the same Rayleigh statistic, as a frequently firing neuron recorded over a much shorter session. In contrast, KDE probabilities implicitly account for the baseline probability of firing (Fig. 1*C*). A sparsely firing neuron recorded over a long session will thus produce a smaller amplitude KDE curve than a frequently firing neuron recorded over a shorter session, for the same number of spikes and same degree of phase relationship. This distinction crucially disambiguates rhythmic neurons—where rhythmicity requires firing at consistent phase bins for a large fraction of consecutive cycles—from neurons that exhibit systematic phase relationships but which spike so rarely as to coincide with a smaller, and more sporadic (e.g., nonconsecutive), fraction of the rhythm’s many cycles. In this regard, the Rayleigh statistic and our modeling approach provide complementary information that together fully captures systematic relationships to LFP rhythms, as well as the likelihood of a neuron’s rhythmicity.

When we applied cross-validated model comparisons between phaseSH and SH models to multiple behavioral contexts and neuron types, we revealed a range of dynamic relationships to theta and low gamma rhythms in the hippocampus. Specifically, we identified changing degrees of CA1 interneuron entrainment to theta and low gamma rhythms across high velocity and odor sampling epochs (Fig. 4), and even changing entrainment over the course of the odor sampling interval at fine (50 ms) time scales (Figs. 5 and 6; Supplemental Fig. S8). Given the dynamism we describe in the hippocampus, and the often highly task-selective responses of hippocampal neurons, we recommend thoughtful consideration of the isolated intervals of interest. Specifically, eliciting many trials with stereotyped behavior is necessary to best leverage the ability of our method to characterize reliable rhythmic entrainment when cells are active and the evolution of rhythmic entrainment over time for particular processing states.

Oscillatory currents prominently manifest within a variety of brain regions. Sensory stimuli can evoke a cascade of oscillatory interactions, which often reflect tight excitatory and inhibitory control of spiking in piriform cortex during the presentation of odors (47), and produce exquisite gamma oscillatory dynamics in visual cortices in response to attended stimuli (48, 49). In motor systems, tight coordination can also be observed throughout the basal ganglia in response to utilized cues (50) but also in traveling waves through motor cortices during movement preparation and execution (51–53). Aberrant oscillatory patterns have been observed in a number of neural disorders [e.g., disrupted gamma in Alzheimer’s disease (54, 55), disrupted β in Parkinson’s disease (56)] and are commonly used as markers of altered interactions and disease progression. However, in all cases, the relationship between these oscillatory currents and cell spiking activity has been poorly understood due to a limited ability to assess spiking relationships to ongoing oscillations at the time scales over which they evolve (as opposed to averages over seconds-long epochs). Our modeling approach can be applied broadly in these contexts to precisely characterize how and when large scale, coordinated currents engage cells to support dynamic information processing.

## SUPPLEMENTAL DATA

10.6084/m9.figshare.16663216 Supplemental Fig. S1: https://doi.org/10.6084/m9.figshare.16663216;

10.6084/m9.figshare.20293863 Supplemental Fig. S2: https://doi.org/10.6084/m9.figshare.20293863;

10.6084/m9.figshare.16663231 Supplemental Fig. S3: https://doi.org/10.6084/m9.figshare.16663231;

10.6084/m9.figshare.16663240 Supplemental Fig. S4: https://doi.org/10.6084/m9.figshare.16663240;

10.6084/m9.figshare.19684068 Supplemental Fig. S5: https://doi.org/10.6084/m9.figshare.19684068;

10.6084/m9.figshare.19617693 Supplemental Fig. S6: https://doi.org/10.6084/m9.figshare.19617693;

10.6084/m9.figshare.19621851 Supplemental Fig. S7: https://doi.org/10.6084/m9.figshare.19621851;

10.6084/m9.figshare.19678359 Supplemental Fig. S8: https://doi.org/10.6084/m9.figshare.19678359.

## GRANTS

This research was partially funded by Frontiers of Innovation Scholars Program, FISP (to L. M. Rangel and T. P. Coleman); Hellman Fellowship (to L. M. Rangel); Kavli Institute for Brain and Mind (to L. M. Rangel, P. D. Rivière, T. P. Coleman, and G. Schamberg); National Institutes of Health Grant R01MH1105142 (A.C. – Andrea Chiba, to T. P. Coleman and L. M. Rangel); National Institutes of Mental Health Grant R03MH120406 (to L. M. Rangel and T. P. Coleman); National Institutes of Mental Health MH052090 (H.E. – Howard Eichenbaum); National Science Foundation Graduate Research Fellowship Program (to P. D. Rivière); National Science Foundation DMS-1042134 (to L. M. Rangel); National Science Foundation Science of Learning Center Grant SBE 0542013, Temporal Dynamics of Learning Center (P. D. Rivière and L. M. Rangel).

## DISCLOSURES

No conflicts of interest, financial or otherwise, are declared by the authors.

## AUTHOR CONTRIBUTIONS

P.D.R., G.S., T.P.C., and L.M.R. conceived and designed research; P.D.R., G.S., and L.M.R. performed experiments; P.D.R. analyzed data; P.D.R., G.S., T.P.C., and L.M.R. interpreted results of experiments; P.D.R. prepared figures; P.D.R., G.S., T.P.C., and L.M.R. drafted manuscript; P.D.R., G.S., T.P.C., and L.M.R. edited and revised manuscript; P.D.R., G.S., T.P.C., and L.M.R. approved final version of manuscript.
